# Identification of natural products as novel ligands for the human 5-HT_2C_ receptor

**DOI:** 10.1007/s41048-018-0047-1

**Published:** 2018-03-09

**Authors:** Yao Peng, Simeng Zhao, Yiran Wu, Haijie Cao, Yueming Xu, Xiaoyan Liu, Wenqing Shui, Jianjun Cheng, Suwen Zhao, Ling Shen, Jun Ma, Ronald J. Quinn, Raymond C. Stevens, Guisheng Zhong, Zhi-Jie Liu

**Affiliations:** 1 National Laboratory of Biomacromolecules, Institute of Biophysics, Chinese Academy of Sciences, Beijing, 100101 China; 2 Institute of Molecular and Clinical Medicine, Kunming Medical University, Kunming, 650500 China; 3 iHuman Institute, ShanghaiTech University, Shanghai, 201210 China; 4 University of Chinese Academy of Sciences, Beijing, 100049 China; 5 School of Life Science and Technology, ShanghaiTech University, Shanghai, 201210 China; 6 College of Pharmacy, Nankai University, Tianjin, 300071 China; 7 High-throughput Molecular Drug Discovery Center, Tianjin Joint Academy of Biotechnology and Medicine, Tianjin, 300457 China; 8 Eskitis Institute for Drug Discovery, Griffith University, Brisbane, QLD 4111 Australia

**Keywords:** GPCR, 5-HT_2C_ receptor, Natural product, Alkaloids

## Abstract

G protein-coupled receptors (GPCRs) constitute the largest human protein family with over 800 members, which are implicated in many important medical conditions. Serotonin receptors belong to the aminergic GPCR subfamily and play important roles in physiological and psychological activities. Structural biology studies have revealed the structures of many GPCRs in atomic details and provide the basis for the identification and investigation of the potential ligands, which interact with and modulate the receptors. Here, an integrative approach combining a focused target-specific natural compound library, a thermal-shift-based screening method, affinity mass spectrometry, molecular docking, and *in vitro* as well as *in vivo* functional assay, was applied to identify (–)-crebanine and several other aporphine alkaloids as initial hits for a human serotonin receptor subtype, the 5-HT_2C_ receptor. Further studies illuminated key features of their binding affinity, downstream signaling and tissue reaction, providing a molecular explanation for the interaction between (–)-crebanine and human 5-HT_2C_ receptor.

## Introduction

G protein-coupled receptors (GPCRs) are cell surface receptors which are responsible for more than 80% of cell signal transduction across cell membranes. They are involved in a wide range of physiological as well as psychological activities and constitute the largest human protein family with over 800 members. GPCRs are implicated in many medical conditions such as heart disease, metabolic diseases, cancer, immune diseases and neurological disorders (Rask-Andersen *et al.* 2014). Drugs targeting GPCRs comprise as much as 33% of all marketed drugs approved by FDA (U.S. Food and Drug Administration) (Santos *et al.* 2017).

Serotonin, or 5-hydroxytryptamine (5-HT), is a neurotransmitter widely found in both the central nervous system (CNS) and the peripheral nervous system. It plays important roles in the functions of brain, gastrointestinal tract, cardiovascular system, and immune cells. In the CNS, serotonergic system regulates mood, perception, memory, food intake, sexual behaviors, and other functions. These physiological roles of serotonin are mediated by serotonin receptors, which are composed of fourteen subtypes in the mammalian system that are further grouped into seven subfamilies (5-HT_1–7_). Except for 5-HT_3_, which functions as a ligand-gated ion channel, all other serotonin receptors belong to the GPCR superfamily (McCorvy and Roth 2015).

Among serotonin receptors, the 5-HT_2C_ receptor belongs to the 5-HT_2_ subfamily. It shares 58% and 55% sequence similarity with the 5-HT_2A_ and 5-HT_2B_ receptors, respectively (Isberg *et al.* 2016). Activation of 5-HT_2C_ receptor is believed to reduce appetite (Halford and Harrold 2012) and cure schizophrenia (Cheng *et al.* 2016). In 2012, lorcaserin, a selective 5-HT_2C_ agonist, was approved by the FDA for the treatment of obesity (Narayanaswami and Dwoskin 2016). Its efficacy in treatment of nicotine addiction is currently being evaluated in clinical trials (Zeeb *et al.* 2015). Moreover, 5-HT_2C_ has been demonstrated as a potential therapeutic target for treatment of mental disorders also (Englisch *et al.* 2016). Agomelatine, antagonist of 5-HT_2C_ receptor, is used for treating depression and schizophrenia (Jacobson 2015).

The ligand-binding pockets of all serotonin receptors are very similar and, therefore, it is difficult to design an inhibitor exhibiting high degree of specificity for a particular serotonin receptor. Most of the marketed drugs targeting serotonin receptors have side effects which arise due to low specificity. For drug candidates targeting the 5-HT_2C_ receptor, achieving high selectivity is very important because non-specific interaction of the drug with the other two 5-HT_2_ receptors causes side effects or toxicities. Unwanted activation of 5-HT_2A_ by drugs targeting the 5-HT_2C_ receptor causes hallucinations (Nichols *et al.* 2002). Similarly, non-specific activation of 5-HT_2B_ leads to valvular heart disease (Connolly *et al.* 1997).

Ligand identification for GPCRs is a tedious, time-consuming and resource intensive process. In the past few years, new assay methods have been developed to explore multidimensional chemical space in a much more efficient manner. These methods range from cell-based (Besnard *et al.* 2012), label-free (*e.g*., Surface Plasmon Resonance; SPR, and Isothermal Titration Calorimetry; ITC), new biosensors (Kroeze *et al.* 2015) to in silico screening (Huang *et al.* 2015), which has significantly increased the success rate of hit identification when compared to traditional methods.

Natural products contain chemical compounds or substances with pharmacological or biological activities, which can be harnessed for therapeutic benefit or treating diseases as exemplified by traditional Chinese medicine (All natural 2007). In the development of modern medicines, natural products are often used as starting points for drug discovery and have been considered as the most important resource for identification of lead compounds due to their diverse molecular architectures and a wide range of bioactivities (Ahn 2016). Consequently, during the past 30 years, natural products have been instrumental in the discovery of more than half of the approved drugs (Newman and Cragg 2016).

Given the challenges involved in GPCR ligand discovery; in particular, the need to screen a large chemical space, one efficient approach for identifying hits would be to design or select focused screening libraries to reduce the workload. In this study, as the 5-HT_2C_ receptor belongs to monoamine type of receptors in the class A of GPCR family, we decided to screen novel ligands from alkaloids containing basic nitrogen atoms. The positively charged nitrogen atom of the ligand was expected to anchor to the highly conserved D^3.32^ of the aminergic receptors. The initial screening was performed using thermal stability assay (Alexandrov *et al.* 2008) against a focused alkaloid library consisting of over 300 chemical components isolated from plants (Shang *et al.* 2010). (–)-Crebanine and several other aporphine alkaloids were identified as potential hits for the 5-HT_2C_ receptor. The affinity mass spectrometry (MS) method was used to validate the hits and measure the binding affinities (Chen *et al.* 2015; Qin *et al.* 2015). The cell-based calcium influx assay was employed to characterize the function of the validated hits. Molecular docking studies coupled with site directed mutagenesis were used to predict the binding sites for the compound. A patch clamp experiment was also utilized to investigate the compound’s physiological effects in neurons.

## Results

### Aporphine alkaloids as potential ligands for 5-HT_2C_ receptor

In order to obtain conformationally homogeneous, thermo-stable and highly pure protein samples for screening and characterization of potential ligand hits, the expression construct of 5-HT_2C_ receptor was optimized as described previously (Lv *et al.* 2016). The final expression construct contains a BRIL (PDB ID 1M6T, MW 11.9 kDa) as a stabilizing fusion partner inserted in receptor’s third intracellular loop (ICL3) between L246 and Q301. Additionally, the N- and C-terminals were truncated by 39 and 65 residues, respectively. The optimized construct was inserted into a pFastbac vector for expression in *Spodoptera frugiperda* (*Sf9*) cells.

**Fig. 1:**
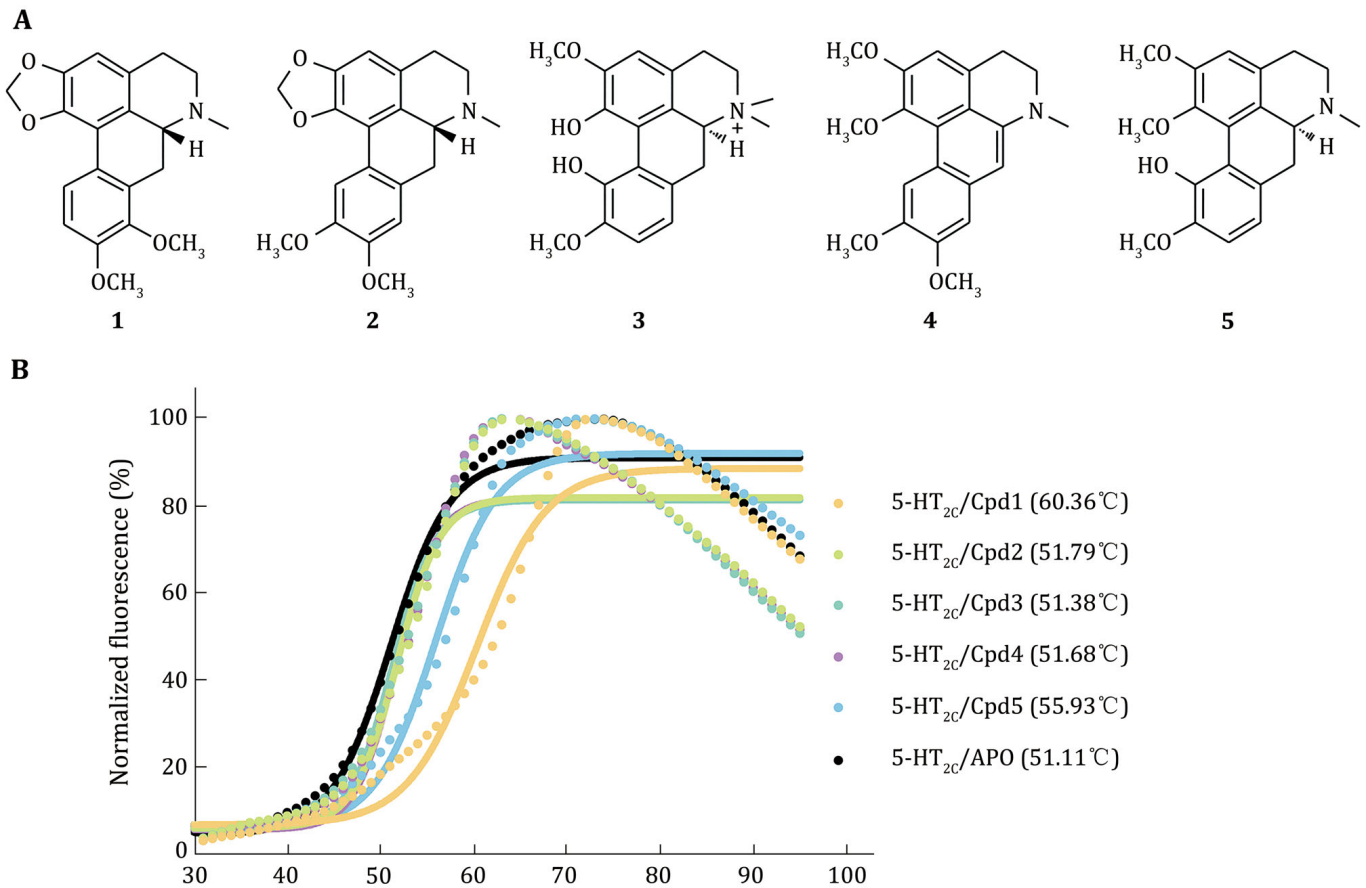
**A** Structures of aporphine alkaloids. The five aporphine alkaloids: (–)-crebanine (**1**), (–)-dicentrine (**2**), (+)-magnoflorine (**3**), didehydroglaucine (**4**), (+)-isocorydine (**5**). **B** Thermo-stability values of aporphine alkalodis. Thermal stability ramping assay of 5-HT_2C_ receptor combine with aporphine alkalodis. The Tm value of the 5-HT_2C_/compound **1** (*yellow trace*) is higher than other combinations, indicating that 5-HT_2C_/compound **1** combination improves the thermostability of 5-HT_2C_ receptor

A small pipetting workstation (Qiagility, Qigen) and a real-time fluorescence quantitative PCR were used to perform the high throughput CPM (the thiol-specific fluorochrome N-[4-(7-diethylamino-4-methyl-3-coumarinyl) phenyl] maleimide) screening (Alexandrov *et al.* 2008). Aporphine alkaloids (1–5) were identified as potential hits for 5-HT_2C_ receptors (Fig. 1A). Among them, (–)-crebanine (**1**) and (+)-isocorydine (**5**) showed more significant binding property to 5-HT_2C_ receptor, with the thermal shift value (the difference between target A and B for melting temperature in CPM assay, ∆Tm) of 9.25 and 4.82 °C, respectively, comparing to apo-protein (Fig. 1B). The other three alkaloids, (–)-dicentrine (**2**), (+)-magnoflorine (**3**), and didehydroglaucine (**4**) showed slight temperature shift (∆Tm < 1.00 °C) in the thermal shift experiments.

### Ligand validation by ultrafiltration-based affinity mass spectrometry analysis

**Fig. 2:**
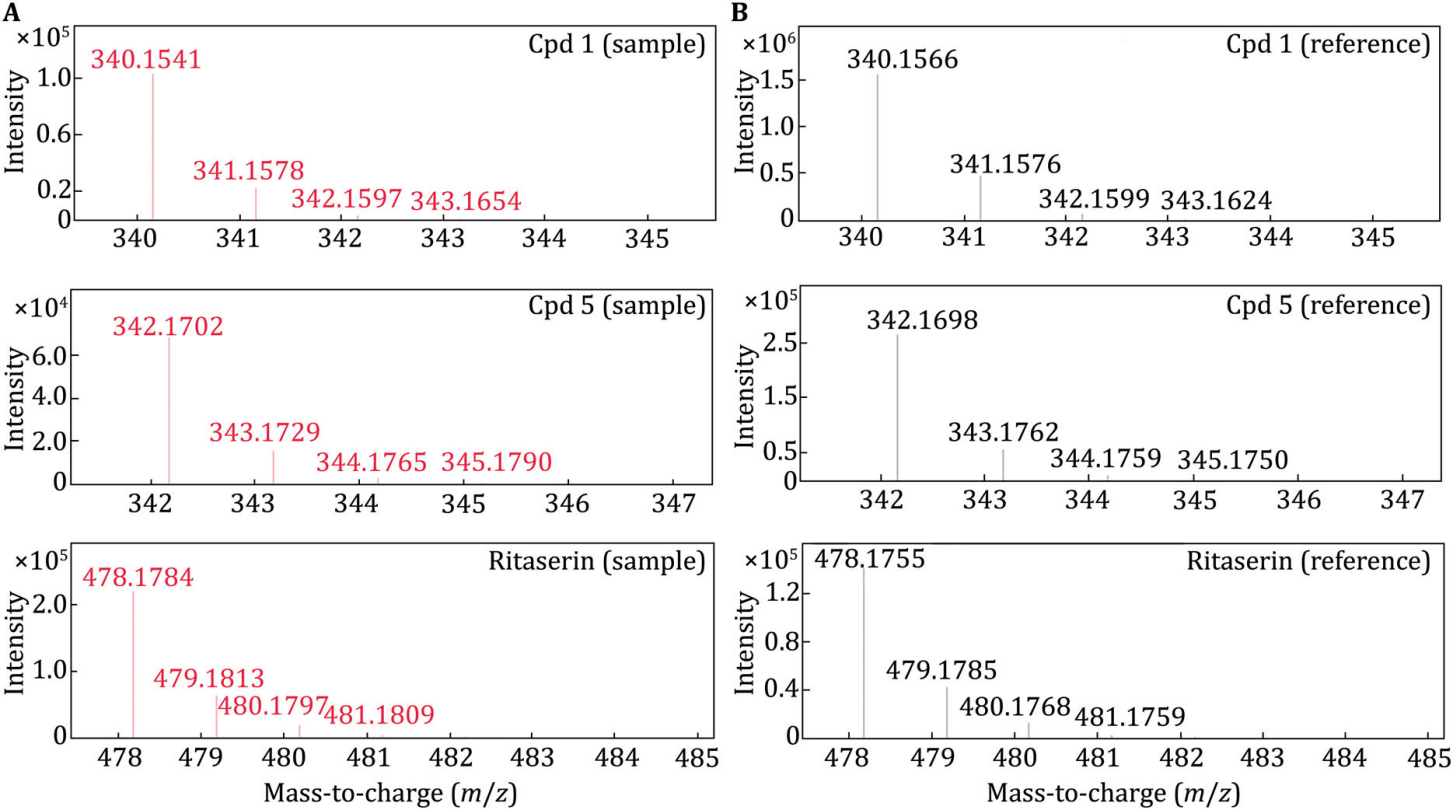
Mass spectra of three compounds detected in the 5-HT_2C_ receptor incubation sample (**A**) and the corresponding reference (**B**). Matching accurate mass and retention time of each ligand with the reference data are required for confident structural assignment

The ultrafiltration-based affinity MS technique has been established to search for ligands, verify binding and estimate affinity of specific ligands for given soluble protein targets (Chen *et al.* 2015; Qin *et al.* 2015). In this study, this technique was adapted to ligand-binding validation for the membrane 5-HT_2C_ receptor. To confirm binding specificity of (–)-crebanine (**1**) and (+)-isocorydine (**5**) to 5-HT_2C_ receptor, a negative control was prepared using another GPCR protein (hydroxycarboxylic acid receptor 2). An S/C ratio referring to the ratio of MS response of a given ligand detected in the 5-HT_2C_ receptor incubation sample versus the control was used to assess specific enrichment of the ligand associated with 5-HT_2C_ receptor. Previous study has shown that ligands with an S/C ratio > 2 are positive binders of the target protein (Chen *et al.* 2015). For (–)-crebanine (**1**) and (+)-isocorydine (**5**), their S/C ratios are significantly above the threshold and very close to a known high affinity 5-HT_2C_ receptor antagonist ritanserin, indicating that they both showed obvious interactions with 5-HT_2C_ receptor (Table 1). The high-resolution mass spectra for both compounds in the protein complex fraction confirmed their structural identification (Fig. 2). Then, a single-point *K*_d_ calculation method (Qin *et al.* 2015) was employed to estimate binding affinity of each ligand to the receptor (Table 1). It turned out that (–)-crebanine (**1**) displayed stronger affinity (*K*_d_ ~ 0.34 μmol/L) than its analog (+)-isocorydine (**5**) (*K*_d_ ~ 11 μmol/L) whereas the affinity of ritanserin (positive control) was in the high nmol/L range.

**Table 1:** S/C ratios and estimated binding affinity of two new ligands and ritanserin

	Compound **1**	Compound **5**	Ritanserin
S/C	9.15 ± 1.43	7.89 ± 0.74	8.39 ± 1.80
*K*_d_ (μmol/L)	0.34 ± 0.07	11.0 ± 0.75	0.37 ± 0.05

For each ligand, S/C and *K*_d_ measurements were represented by the average values and standard deviations from experimental replicates (*n* = 4)

### Calcium influx assay characterization of the potential hits

**Fig. 3:**
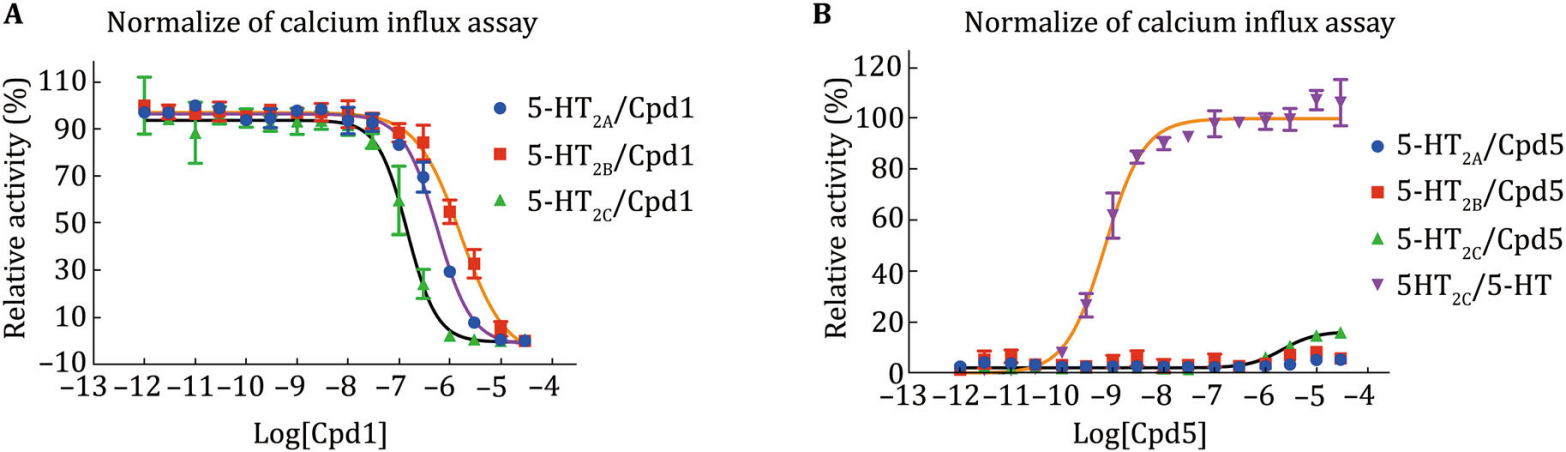
Fluorescence-based calcium mobilization measurement of 5-HT_2A/2B/2C_ mediated G_q_ inhibition/activation by (–)-crebanine (**1**) and (+)-isocorydine (**5**), all in HEK 293 derived cells. **A** Normalized inhibition of compound **1** on human cloned 5-HT_2A/2B/2C_ receptor-mediated Gq signaling. 3 nmol/L of 5-HT was used to induce initial activation. The 5-HT_2C_ receptor shows the lowest *IC*_50_ among the tested receptors. **B** Normalized activation of compound **5** on human cloned 5-HT_2A/2B/2C_ receptor-mediated G_q_ signaling. Compound **5** shows partial agonism against 5-HT_2C_ receptor

The 5-HT_2C_ receptor mainly couples to G_αq_ proteins. The activated 5-HT_2C_ receptor transmits the signals from the extracellular to the intercellular side using DAG/IP_3_ (diacyl glycerol/inositol 1,4,5-trisphosphate) as its second massagers (Hannon and Hoyer 2008). IP_3_ stimulates the endoplasmic reticulum to release calcium ions into the cytoplasm, which causes the increase of calcium concentration in cytosol. Therefore, calcium mobilization assay is commonly applied to understand how unknown small molecule ligands of 5-HT_2C_ receptor modulate cellular signal transductions. In this case, Fluo-4 Direct dye was used as a fluorescence indicator to detect calcium flux in G protein-coupled receptor expressed cells. The fold of maximum response over average of basal reading is plotted against compound concentration to determine the potency of the compounds (Fig. 3). (–)-Crebanine (**1**) displayed antagonism for 5-HT_2_ receptors (Fig. 3A). When 5-HT_2A/2B/2C_ receptors were activated by 3 nmol/L of 5-HT, (–)-crebanine (**1**) inhibited the activation at *IC*_50_ at 564, 1693 and 149 nmol/L, respectively. (–)-Crebanine (**1**) showed higher efficacy with the *IC*_50_ value in 5-HT_2C_ receptor compared to 5-HT_2A_ and 5-HT_2B_ receptors. (–)-Crebanine (**1**)’s analog (+)-isocorydine (**5**) showed very weak partial agonism towards 5-HT_2C_ receptor (*E*_max_ = 16.6% of the effect of 1 μmol/L 5-HT) (Fig. 3B). While 5-HT_2C_ receptor was activated (*EC*_50_ = 2075 nmol/L), 5-HT_2A/2B_ receptors remained inactive with the addition of up to 30,000 nmol/L (+)-isocorydine (**5**). Hence, (–)-crebanine (**1**)’s analog, (+)-isocorydine (**5**), showed different pharmacology characters towards 5-HT_2C_ receptor.

### Identification of key interactions of (–)-crebanine with 5-HT_2C_ receptor

**Fig. 4:**
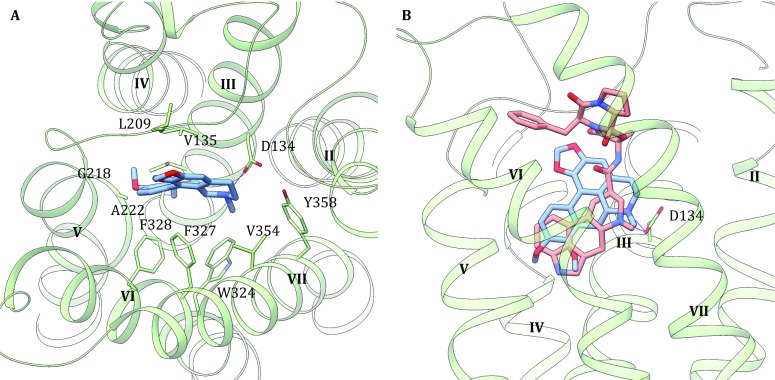
Docked pose of (–)-crebanine in 5-HT_2C_ model. **A** Ligand-receptor interactions. The key residues were labeled and showed in sticks. **B** Superposition with ergotamine in 5-HT_2B_ crystal structure

Binding mode of (–)-crebanine (**1**) in 5-HT_2C_ receptor model (built based on the crystal structure of 5HT_2B_ receptor, PDB ID: 4IB4) is predicted by molecular docking (Fig. 4A). In the previous studies, the crystal structures of 5-HT_1B/2B_ in complex with ergotamine and 5-HT2B with lysergic acid diethylamide (LSD) revealed similar orthosteric ligand-binding cavities defined by residues from helixes III, V, VI, VII, and ECL2 (Wacker *et al.* 2013, 2017; Wang *et al.* 2013). The binding pocket is embedded deep in the 7TM core of the receptor and (–)-crebanine (**1**) partially overlaps with the ergoline rings of ergotamine bound to 5-HT_1B/2B_ receptor structures. Some key interactions are in common for (–)-crebanine and ergotamine: A salt bridge is formed between the positively charged nitrogen of (–)-crebanine (**1**) and the carboxylate of Asp134^3.32^ (Venkatakrishnan *et al.* 2013) (Fig. 4B), which is fully conserved in 5-HT and other monoamine receptors. (–)-Crebanine (**1**) also forms π–π interaction with a benzene ring to Phe327^6.51^, which resembles similar feature of ergotamine bound to 5-HT_1B_ and 5-HT_2B_ receptors. Due to the different shapes of aporphine and ergoline rings, (–)-crebanine also extends to space close to the entrance and forms hydrophobic interactions to Leu209 and Phe214 on ECL2. Hydrophobic interactions between (–)-crebanine (**1**) and Val135^3.33^, Thr139^3.37^, Gly218^5.42^, Ala222^5.46^, Trp324^6.48^, Val354^7.39^, and Tyr358^7.43^ are also predicted. The different binding affinities and functions of (–)-crebanine (**1**) (antagonist) and (+)-isocorydine (**5**) (weak partial agonist) may due to the difference in chirality of the carbon atom or substitution groups in the aporphine scaffold. In this predicted binding mode, the orientation of aporphine scaffold is the same to previously published docking results of dihydrofuroaporphine in 5HT_1A_ (Yuan *et al.* 2016).

Based on the binding poses predicted by molecular docking, single-point mutations were designed to test their impact on ligand binding. Mutations of several residues include the highly conserved Asp134^3.32^, which is known to cause 5-HT_1B_ or 5-HT_2B_ receptors to abolish the monoamine ligand binding as reported in the literature (Wang *et al.* 2013). In this study, residues forming hydrophobic interactions with the substitution groups on the aporphine scaffold of (–)-crebanine (**1**) were selected for mutation as well and they are Thr139^3.37^, Gly218^5.42^, Ala222^5.46^, and Leu209^ECL2^. Effects of those mutations were tested with the CPM assay and the affinity MS assay (Table 2). In the affinity MS experiment, relative ligand-binding capacity (represented by binding %) to different mutants and the wild-type receptor was compared. Among the six mutations tested, five of them reduced the binding of (–)-crebanine (**1**) by more than 40% relative to binding to the wild-type receptor, displaying the importance of hydrophobic interactions. Mutations Gly218^5.42^Ala/Ser impacted the binding of (–)-crebanine the most, causing the Tm values decreased over 6.0 °C and ligand-binding capacity reduced by over 60%. These results fit the predicted compact interactions of (–)-crebanine (**1**) to Gly218^5.42^. Thr139^3.37^ is highly conserved residue in serotonin receptors, and Thr139^3.37^Ala led to the Tm value decrease and ligand potency lost. Leu209^ECL2^Phe also has significant influence on the binding of (–)-crebanine (**1**), supporting our prediction that (–)-crebanine (**1**) forms hydrophobic interactions to residues on ECL2. Ala222^5.46^Phe has a similar effect to Thr139^3.37^ and Leu209^ECL2^Phe, while Ala222^5.46^Val is the only mutation having little effect on the thermostability of (–)-crebanine (**1**) bound 5-HT_2C_ receptor (Tm value decreased by 0.38 °C) and ligand-binding capacity to the mutant inferred from the affinity MS assay. Different effects of mutations Ala222^5.46^Phe/Val show that there is some space in the pocket around this site, but too bulky side chain collides with (–)-crebanine (**1**).

**Table 2:** Mutations validation on the ligand binding pocket using thermostability and affinity MS assay

Mutants	(–)-Crebanine/CPM (*n* = 2) (°C)	(–)-Crebanine/MS (*n* = 2)^a^ (%)
WT	59.93	27.57
G218^5.42^A	53.92	9.82
G218^5.42^S	53.24	6.04
A222^5.46^V	59.55	25.84
A222^5.46^F	55.70	15.87
T139^3.37^A	54.63	14.64
L209^ECL2^F	55.46	13.22

^a^Relative ligand binding (%) was calculated by the MS response of the ligand released from a specific mutant divided by the MS response of the total ligand present in the incubation sample

### Crebanine attenuated the spontaneous synaptic current amplification induced by 5-HT

**Fig. 5:**
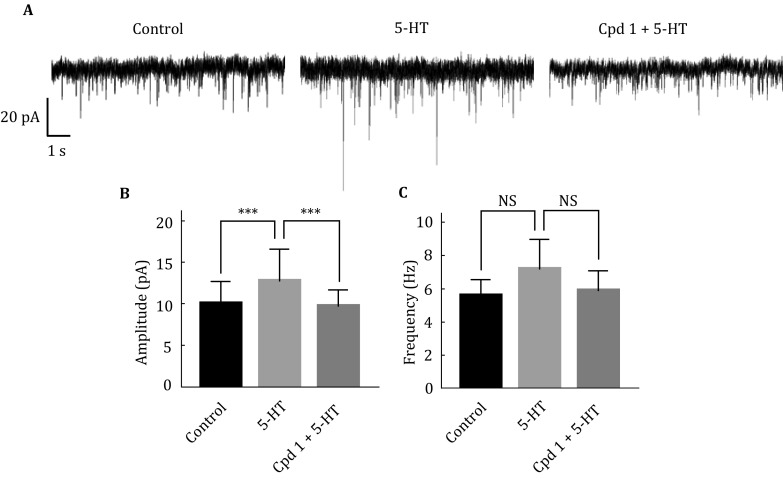
Crebanine attenuated the SPSC amplification induced by 5-HT. **A** Representative traces of SPSC in control condition (*left*), and in response to 5-HT, with (*middle*) or without (*right*) (–)-crebanine. Amplitude histograms (**B**) and frequency histograms (**C**) of SPSC (***p < 0.001)

5-HT receptors play important roles in central and peripheral nervous systems. Malfunctions of these receptors have been linked to many neural disorders such as depression and psychosis. The effect of (–)-crebanine on changes in 5-HT induced synaptic transmission was evaluated by electrophysiological recording of mouse brain slices (Jang *et al.* 2012). The spontaneous synaptic current (SPSC) of cortical pyramidal neurons was recorded in whole cell mode. The SPSC was amplified after 5-HT treatment at the concentration of 10 μmol/L, while the effect of 5-HT induced SPSC was significantly attenuated in the presence of 30 μmol/L (–)-crebanine (**1**) incubation (Fig. 5).

## Discussion

Natural products contain a variety of components like alkaloids, peptides, lipids, glucosides, *etc.*. These diverse components are perfect candidates for serving as ligands to modulate the activity of G protein-coupled receptors. Furthermore, a co-evolutional relationship has been noted between receptors and plants. This is supported by the fact that many endogenous ligands are related to phyto-compounds and there is a certain degree of overlap between the chemical spaces of endogenous ligands and components of natural products. Therefore, screening of focused libraries containing a sub-set of natural products that show chemical similarity to the known ligands of the target receptors has the potential to significantly increase the success rate of discovery of new ligands. Serotonin (5-HT) shares its scaffold with many alkaloids from plants. To identify novel ligands for 5-HT_2C_ receptor, we selected and screened a focused natural products-based library enriched in alkaloids. A potential hit, (–)-crebanine (**1**), increased the Tm of 5-HT_2C_ receptor by 9.25 °C during thermal denaturation assays. Ultrafiltration-based affinity mass spectrometry validated the interaction between (–)-crebanine (**1**) and 5-HT_2C_ receptor, and estimated the affinity of the ligand for the receptor (*K*_d_ ~ 0.34 μmol/L). Using cell-based calcium influx assay, we identified (–)-crebanine (**1**) to be an antagonist of 5-HT_2C_ receptor. Furthermore, we predict its binding pose in 5-HT_2C_ receptor by molecular docking and several key interactions of the ligand with the protein are proposed. The subsequent mutagenesis and binding experiments confirmed the predicted binding pose. In order to find out if (–)-crebanine (**1**) plays a role in modulating the function of 5-HT receptors at tissue level, we recorded the electrical potential across the membrane of neurons from mice brain slices. (–)-Crebanine (**1**) inhibited the excitability caused by 5-HT in the brain slices, implying that (–)-crebanine (**1**) is effective in inhibiting the activity of 5-HT receptors and counteracting the excitatory role of 5-HT in the central nervous system of mice.

In summary, we have identified (–)-crebanine (**1**) as an antagonist for 5-HT_2A/2B/2C_ receptors. The *IC*_50_ value of the compound was the lowest (149 nmol/L) for 5-HT_2C_ receptor, suggesting some specificity in targeting the type of receptor. Structure-guided optimization of the crebanine scaffold is likely to further increase the receptor specificity. This is important because inhibition of 5-HT_2C_ receptor activity has been shown to cure depression, schizophrenia and drug addiction. Results of our studies provide a framework for developing 5-HT_2C_ receptor-specific antipsychotic drug. We show that natural products can be a viable source of novel ligands for 5-HT_2C_ receptor. A comprehensive approach to screen and characterize novel ligands of GPCRs from the natural product library is also described in this study. We believe that the experimental protocols and analytical methods are general and could be used to facilitate biochemical and pharmacological studies for discovery of novel ligands targeting other GPCRs.

## Experimental section

### Compounds library preparation

(–)-Crebanine (compound **1**) and (–)-dicentrine (compound **2**) were isolated from *A. scholaris* as previously described (Shang *et al.* 2010). Compounds (**3**–**5**) were purchased from Herbpurify Co., Ltd., Chengdu, China. Briefly, organic compounds from dried and powdered leaves of *A. scholaris* were extracted with ethanol and the solvent was evaporated under vacuum to obtain an extract. The extract was dissolved in 1% HCL and the constituents of the solution were considered as the alkaloid fraction. The solution was basified using ammonia water and extracted with ethyl acetate. The alkaloid extract was subjected to silica gel chromatography and eluted with chloroform–methanol (30:1–1:1) mixture in six fractions (I–VI). (–)-Crebanine and (–)-dicentrine were purified from fraction IV after column chromatography over silica gel (chloroform-acetone) and reverse phase RP_18_ (methanol–water). The structures were validated by ^1^H NMR spectrum analysis.

(–)-Crebanine (**1**) was obtained as colorless powder. ^1^H NMR (CDCl_3_, 500 MHz): *δ* 7.80 (1H, d, *J* = 8.5 Hz, H-11), 6.88 (1H, d, *J* = 8.5 Hz, H-10), 6.53 (1H, s, H-3), 6.06 (1H, d, *J* = 1.4 Hz, -OCH_2_O-), 5.91 (1H, d, *J* = 1.4 Hz, -OCH_2_O-), 3.90 (3H, s, -OCH_3_), 3.81 (3H, s, -OCH_3_), 3.67 (1H, dd, *J* = 14.0, 4.0 Hz, H-7), 3.12 (1H, m, H-6a), 3.05 (2H, m, H-4), 2.63 (1H, dd, *J* = 16.0, 3.0 Hz, H-5), 2.59 (3H, s, -NCH_3_), 2.52 (1H, dd, *J* = 16.0, 3.0 Hz, H-5), 2.30 (1H, dd, *J* = 14.5, 14.0 Hz, H-7)(Bartley *et al.* 1994).

(–)-Dicentrine (**2**) was obtained as colorless powder. ^1^H NMR (CDCl_3_, 500 MHz): *δ* 7.67 (1H, s, H-11), 6.78 (1H, s, H-10), 6.52 (1H, s, H-3), 6.08 (1H, d, *J* = 1.4 Hz, -OCH_2_O-), 5.93 (1H, d, *J* = 1.4 Hz, -OCH_2_O-), 3.92 (3H, s, -OCH_3_), 3.92 (3H, s, -OCH_3_), 3.09 (1H, dd, *J* = 14.0, 4.5 Hz, H-7), 3.12 (1H, m, H-6a), 3.05 (2H, m, H-4), 2.67 (1H, dd, *J* = 16.0, 3.0 Hz, H-5), 2.56 (3H, s, -NCH_3_), 2.62 (1H, dd, *J* = 16.0, 3.0 Hz, H-5), 2.53 (1H, dd, *J* = 14.5, 14.0 Hz, H-7) (Shang *et al.* 2010).

(+)-Magnoflorine (**3**) was obtained as brown powder. ^1^H NMR (DMSO-*d*_*6*_, 400 MHz): *δ* 6.60 (1H, d, *J* = 8.0 Hz, H-9), 6.36 (1H, d, *J* = 8.0 Hz, H-8), 6.51 (1H, s, H-3), 4.37 (1H, d, *J* = 11.5 Hz, H-6a), 3.69 (3H, s, -OCH_3_), 3.66 (3H, s, -OCH_3_), 3.67 (1H, m, overlap, H-5), 3.60 (1H, m, H-5), 3.33 (3H, H, -NCH_3_), 3.12 (1H, dd, *J* = 14.0, 13.0 Hz, H-4), 3.11 (1H, dd, *J* = 13.0, 3.5 Hz, H-7), 2.90 (3H, s, -NCH_3_), 2.82 (1H, dd, *J* = 14.0, 3.0 Hz, H-5), 2.61 (1H, dd, *J* = 14.5, 13.0 Hz, H-7) (Yin *et al.* 2016).

Didehydroglaucine (**4**) was obtained as light greenish powder. ^1^H NMR (DMSO-*d*_*6*_, 400 MHz): *δ* 8.91 (1H, s, H-11), 7.18 (1H, s, H-8), 7.18 (1H, s, H-7), 6.61 (1H, s, H-3), 3.94 (3H, s, -OCH_3_), 3.87 (3H, s, -OCH_3_), 3.86 (3H, s, -OCH_3_), 3.81 (3H, s, -OCH_3_), 3.29 (1H, t, *J* = 6.5 Hz, H-4), 3.20 (1H, t, *J* = 6.5 Hz, H-7), 2.99 (3H, s, -NCH_3_)(Xu *et al.* 2002).

(+)-Isocorydine (**5**) was obtained as colorless crystal. ^1^H NMR (DMSO-*d*_*6*_, 400 MHz): *δ* 8.64 (1H, s, -OH), 6.98 (1H, s, H-3), 6.97 (1H, d, *J* = 8.0 Hz, H-8), 6.88 (1H, d, *J* = 8.0 Hz, H-9), 4.06 (1H, m, H-6a), 3.86 (3H, s, -OCH_3_), 3.80 (3H, s, -OCH_3_), 3.65 (3H, s, -OCH_3_), 3.36 (1H, m, H-7), 3.35 (1H, m, H-5), 3.05 (2H, m, H-4), 2.99 (1H, m, H-5), 2.98 (1H, m, H-7), 2.59 (3H, s, overlap, -NCH_3_)(Zhong *et al.* 2015).

### Cloning

The ∆N-5-HT_2C_-BRIL-∆C DNA was codon optimized, synthesized by DNA2.0 and subcloned into a modified pFastBac1 vector (Invitrogen). The construct had the following features: (1) Residues of the third intracellular loop of the wild-type human 5-HT_2C_ receptor were replaced with Ala1-Leu106 of BRIL; (2) N-terminal residues before and including the glycosylation site and C-terminal residues after the helix 8 of 5-HT_2C_ receptor were truncated. An apocytochrome b_562_ RIL (BRIL) gene from *E. coli*, with M7 W, H102I, and R106L mutations is referred to as BRIL.

The vector designated as pFastBac1-830220 contained an expression cassette with a haemagglutinin (HA) signal sequence followed by a FLAG tag at the N-terminus, and a PreScission protease site followed by a 10× His tag at the C-terminus (Lv *et al.* 2016).

### Virus generation and expression

High-titer recombinant baculovirus (>10^9^ viral particles per ml) was obtained using the Bac-to-Bac Baculovirus Expression System (Invitrogen). Recombinant baculovirus was generated by transfecting 5–10 μg of recombinant bacmid into 2.5 ml *Spodoptera frugiperda* (*Sf9*) cells at a density of 10^6^ cells per ml using 5 μl of FuGENE HD Transfection Reagent (Promega) and Transfection Medium (Expression Systems). After 4 days of shaking at 27 °C, P0 viral stock with ~10^9^ virus particles per ml was harvested and used to generate high-titer baculovirus stock. Viral titers were determined by flow-cytometric analysis of cells stained with gp64-PE antibody (Expression Systems) (Hanson *et al.* 2007). Expression of the 5-HT_2C_ receptor was carried out by infection of *Sf9* cells at a cell density of 2–3 × 10^6^ cells/ml with P1 virus stock at multiplicity of infection (MOI) of five. Cells were harvested by centrifugation at 48 h post-infection and stored at −80 °C until further use.

### Membrane purification

Insect cell membranes were disrupted by thawing frozen cell pellets in a hypotonic buffer containing 10 mmol/L HEPES, pH 7.5, 10 mmol/L MgCl_2_, 20 mmol/L KCl and EDTA-free complete protease inhibitor cocktail tablets (Roche). Extensive washing of the isolated raw membranes was performed by repeated centrifugation in the same hypotonic buffer (two times), and then in a high osmotic buffer containing 1.0 mol/L NaCl, 10 mmol/L HEPES, pH 7.5, 10 mmol/L MgCl_2_, 20 mmol/L KCl and EDTA-free complete protease inhibitor cocktail tablets (three times), to remove soluble and membrane-associated proteins. Purified membranes were directly flash-frozen in liquid nitrogen and stored at −80 °C until further use.

### Protein purification

Purified membranes were resuspended in buffer containing 10 mmol/L HEPES, pH 7.5, 10 mmol/L MgCl_2_, 20 mmol/L KCl, 150 mmol/L NaCl, and EDTA-free complete protease inhibitor cocktail tablets (Roche). Prior to solubilization, membranes were equilibrated at 4 °C and incubated for 30 min in the presence of 2 mg/mL iodoacetamide (Sigma). Membranes were then solubilized in 50 mmol/L HEPES, pH 7.5, 150 mmol/L NaCl, 1% (*w*/*v*) n-dodecyl-β-d-maltopyranoside (DDM, Anatrace), 0.2% (*w*/*v*) cholesteryl hemisuccinate (CHS, Sigma) and EDTA-free complete protease inhibitor cocktail tablets (Roche) for 2 h at 4 °C. Unsolubilized material was removed by centrifugation at 35,000 r/min for 30 min, and buffered imidazole and NaCl were added to the supernatant to adjust concentrations to 20 and 800 mmol/L, respectively. Proteins were bound to TALON IMAC resin (Clontech) overnight at 4 °C. The resin was then washed with 10 column volumes (cv) of Wash Buffer I (50 mmol/L HEPES, pH 7.5, 800 mmol/L NaCl, 0.1% (*w*/*v*) DDM, 0.02% (*w*/*v*) CHS, 20 mmol/L imidazole, 10% (*v*/*v*) glycerol), followed by 5 cv of Wash Buffer II (50 mmol/L HEPES, pH 7.5, 150 mmol/L NaCl, 0.05% (*w*/*v*) DDM, 0.01% (*w*/*v*) CHS, 10% (*v*/*v*) glycerol). Proteins were eluted in 5 cv of Wash Buffer II + 250 mmol/L imidazole. Protein purity and monodispersity were tested by SDS-PAGE and analytical size-exclusion chromatography (aSEC).

### Thermal stability assay

Purified 5-HT_2C_ receptor protein was thoroughly mixed with CPM fluorescent dyes. Protein solution containing the fluorescent dyes and compounds was dispensed into 0.2-ml tubes using a pipetting workstation. The tubes were incubated at 4 °C for 1 h prior to thermal denaturation. As the temperature was ramped from 25 to 95 °C, the protein began to unfold, exposing the cysteine residues that interact with the dye. Fluorescence signals, wavelength 387 nm (excitation) and 463 nm (emission), were monitored (Alexandrov *et al.* 2008). Analysis of 72 different samples was performed in parallel. The thermal denaturation of the samples was evaluated using the scatter plot curve, where the horizontal coordinates represented temperature and the vertical coordinates indicated normalized fluorescence intensity recorded for a particular temperature. Tm was obtained by fitting the data with a Boltzmann sigmoidal function using Prism (GraphPad Software).

### Ligand-binding validation by affinity mass spectrometry analysis

The purified apo 5-HT_2C_ receptor protein was incubated with each pure ligand (compounds **1**, **5** or ritanserin) at a final concentration of 500 nmol/L (protein) and 250 nmol/L (ligand) at 4 °C for 60 min. Individual 5-HT_2C_ mutants were incubated with compound **1** under the same condition. Then, the incubated sample (~1 μg) was filtered through 50 kDa MW cutoff ultrafiltration membrane (Sartorius, Germany) by centrifugation at 13,000 *g* for 10 min at 4 °C in the buffer containing 150 mmol/L ammonium acetate, 0.02% (*w*/*v*) DDM and 0.004% (*w*/*v*) CHS. Buffer exchange was repeated once. The protein complexes retained on the ultrafiltration membrane were transferred to a new centrifugal tube. The ligand was dissociated from the complexes with 90% methanol and separated from the denatured protein by centrifugation at 13,000 *g* for 20 min at 25 °C. Another purified GPCR protein (hydroxycarboxylic acid receptor 2) underwent the same process to serve as a negative control. The supernatant was dried out in speed vacuum, reconstituted in 50% methanol, diluted by tenfold prior to LC–MS analysis using Agilent 6530 TOF equipped with an Agilent 1260 HPLC system. The compound was eluted with 85% acetonitrile/0.1% formic acid from Eclipse Plus C18 column (2.1 mm × 100 mm, 3.5 μm, Agilent, USA) at a flow rate of 0.4 ml/min. Full-scan mass spectra were acquired in the range of 100–1000 *m*/*z* on Agilent 6530 TOF with ESI source settings: voltage 3000 V, gas temperature 350 °C, fragmentor 150 V.

Four experimental replicates were prepared for each pair of the test ligand and the negative control. LC–MS chromatograms for specific ligands were extracted using MassHunter software (Agilent, USA) based on the accurate mass measurement with a tolerance of 15 ppm and also matching RT of the reference compound. MS responses are represented by the peak heights of the corresponding extracted ion chromatograms. S/C ratios refer to the ratio of MS response of a specific ligand detected in the 5-HT_2C_ incubation sample versus the control. A single-point *K*_d_ calculation method established earlier for binding evaluation of pure ligands or simple ligand mixtures was then employed to estimate the affinity of each ligand to the receptor (Qin *et al.* 2015). Relative ligand-binding capacity (represented by binding %) to different mutants was calculated by the MS response of the ligand released from a specific mutant divided by the MS response of the total ligand present in the incubation sample.

### Ca^2+^ mobilization assay

HEK 293T cells stably transfected with 5-HT_2A/2B/2C_ receptor were seeded in 384-well plates at a density of 15,000 cells/well in DMEM containing 1% dialyzed FBS 8 h before the calcium flux assay. After removing medium, cells were then incubated (20 μl/well) for 1 h at 37 °C with Fluo-4 Direct dye (Invitrogen) reconstituted in FLIPR buffer (1× HBSS, 2.5 mmol/L probenecid, and 20 mmol/L HEPES, pH 7.4). After the dye loaded, cells were placed in a FLIPR^TETRA^ fluorescence imaging plate reader (Molecular Devices); drug dilutions, prepared at 3× final concentration in FLIPR buffer and aliquotted into 384-well plates, were also added to the FLIPR^TETRA^. The fluidics module and plate reader of the FLIPR^TETRA^ were programmed to read baseline fluorescence for 10 s (1 read/s), then to add 10 μl of drug/well and to read for 6 min (1 read/s). Fluorescence in each well was normalized to the average of the first 10 reads (*i.e.*, baseline fluorescence). Then, the maximum-fold increase, which occurred within 60 s after drug addition, over baseline fluorescence elicited by vehicle or drug was determined. For positive allosteric modulator and antagonist candidates test, 5-HT EC20 (0.1 nmol/L) and EC80 (3 nmol/L) were used, respectively, to activate receptor.

### Modeling of 5-HT_2C_ in complex with crebanine

Modeling of receptor–ligand complexes was carried out with Schrodinger Suite 2015-4. Homology model of 5-HT_2C_ receptor was built based on the crystal structure of 5-HT_2B_ (PDB entry: 4IB4) using the Advanced Homology Modeling tool. Processing of the protein structure was performed with the Protein Preparation Wizard. 3D structures of the compounds were first generated using the LigPrep tool, then optimized by quantum mechanics in B3LYP/6-31G** level using Jaguar 9.0. The complex structures were generated in three steps: (1) Molecular docking of the compounds into 5-HT_2C_ homology model using Glide 6.9; (2) Structural refinements allowing movement of the compounds and protein atoms within 5 Å using Prime 4.2; (3) Re-scoring the binding modes of compounds in the receptors by the extra-precision score using Glide 6.9.

### Brain slicing preparation

Cortical slices from ICR mouse of both genders (15–21 days) were prepared for electrophysiological recording. In brief, the animals were decapitated and brains were placed in cold sucrose artificial cerebrospinal fluid (sucrose-ASCF, in mmol/L: sucrose 213, KCl 2.5, NaH_2_PO_4_ 1.25, NaHCO_3_ 26,* D*-glucose 10, MgSO_4_ 2, CaCl_2_ 2, pH 7.4, 0–4 °C, saturated with 95% O_2_ and 5% CO_2_). The brains were cut into 280 or 300 μm slices with a vibratome (Leica VT1200S, Germany); slices were then incubated with ACSF (in mmol/L: NaCl 126, KCl 2.5, NaH_2_PO_4_ 1.25, NaHCO_3_ 26,* D*-glucose 25, MgSO_4_ 2, CaCl_2_ 2, pH 7.4, saturated with 95% O_2_ and 5% CO_2_) at room temperature for 1 h.

### Electrophysiological recordings

A whole-cell patch clamp technique was used. One brain slice was transferred into the recording chamber, continually perfused with oxygenated ACSF and viewed under a DIC microscope (60× water immersion lens, Olympus, Japan). Activity of cortical pyramidal neurons was recorded using an amplifier (HEKA EPC 10 USB, Germany) in the voltage clamp mode. The electrode puller (Sutter P-1000, USA) was used to make electrodes with the resistance at 10–13 MΩ when filled with the pipette solution (in mmol/L: potassium gluconate 140, KCl 3, MgCl_2_ 2, HEPES 10, EGTA 0.2, Na_2_ATP 2, pH 7.25). Drug treatments were performed by switching the perfusion solution with different drugs (5-HT, compound **1**, compound **1** and 5-HT). To evaluate the effect of **1** against 5-HT, the brain slices were pretreated by **1** for 15–20 min.

## Abbreviations

5-HT: 5-hydroxytryptamine
CNS: Central nervous system
CPM: The thiol-specific fluorochrome N-[4-(7-diethylamino- 4-methyl-3-coumarinyl) phenyl] maleimide
GPCR: G protein-coupled receptor
ICL3: Intracellular loop 3
LSD: Lysergic acid diethylamide
MS: Mass spectrometry
*Sf9*: *Spodoptera frugiperda* (an insect cell line)
SPSC: Spontaneous synaptic current
